# APOBEC3G-Mediated G-to-A Hypermutation of the HIV-1 Genome: The Missing Link in Antiviral Molecular Mechanisms

**DOI:** 10.3389/fmicb.2016.02027

**Published:** 2016-12-19

**Authors:** Ayaka Okada, Yasumasa Iwatani

**Affiliations:** ^1^Department of Microbiology and Immunology, Laboratory of Infectious Diseases, Clinical Research Center, National Hospital Organization Nagoya Medical CenterNagoya, Japan; ^2^Department of AIDS Research, Nagoya University Graduate School of MedicineNagoya, Japan

**Keywords:** HIV-1, APOBEC3G, antiviral mechanisms, deamination, reverse transcription

## Abstract

APOBEC3G (A3G) is a member of the cellular polynucleotide cytidine deaminases, which catalyze the deamination of cytosine (dC) to uracil (dU) in single-stranded DNA. These enzymes potently inhibit the replication of a variety of retroviruses and retrotransposons, including HIV-1. A3G is incorporated into *vif*-deficient HIV-1 virions and targets viral reverse transcripts, particularly minus-stranded DNA products, in newly infected cells. It is well established that the enzymatic activity of A3G is closely correlated with the potential to greatly inhibit HIV-1 replication in the absence of Vif. However, the details of the underlying molecular mechanisms are not fully understood. One potential mechanism of A3G antiviral activity is that the A3G-dependent deamination may trigger degradation of the dU-containing reverse transcripts by cellular uracil DNA glycosylases (UDGs). More recently, another mechanism has been suggested, in which the virion-incorporated A3G generates lethal levels of the G-to-A hypermutation in the viral DNA genome, thus potentially driving the viruses into “error catastrophe” mode. In this mini review article, we summarize the deaminase-dependent and deaminase-independent molecular mechanisms of A3G and discuss how A3G-mediated deamination is linked to antiviral mechanisms.

## Introduction

The human apolipoprotein B mRNA editing enzyme catalytic subunit 3 (A3) family of cellular polynucleotide cytidine deaminases comprises seven members (A, B, C, D, F, G, and H) that catalyze the conversion of cytosine (dC) to uracil (dU) on single-stranded DNA (ssDNA; Kitamura et al., 2011). These enzymes, particularly A3G, exhibit potent antiviral activity against retrotransposons and retroviruses, including HIV-1 (Sheehy et al., 2002; Simon et al., 2005; Bogerd et al., 2006; Muckenfuss et al., 2006; Okeoma et al., 2007; Hultquist et al., 2011; Chaipan et al., 2013). However, the HIV-1 accessory protein viral infectivity factor (Vif), antagonizes the A3G-mediated host defense system, thereby promoting the propagation of HIV-1 in human cells (Sheehy et al., 2002; Conticello et al., 2003). Vif recruits A3G into the E3 ubiquitin ligase complex, including Cullin5, ElonginB/C, and core binding factor subunit beta (CBFβ), and promotes A3G degradation through the ubiquitin-proteasome pathway (Yu et al., 2003; Yu Y. et al., 2004; Salter et al., 2012; Guo et al., 2014). Thus, in the absence of Vif, A3G is incorporated into HIV-1 virions from their viral producers and exerts its antiviral activity in newly infected cells (Sheehy et al., 2002; Anderson and Hope, 2008). The A3G incorporation depends on its binding affinity to the viral nucleocapsid (NC) domain of Gag and/or to the viral/non-viral RNAs (Cen et al., 2004; Luo et al., 2004; Svarovskaia et al., 2004; Burnett and Spearman, 2007; Strebel and Khan, 2008; Apolonia et al., 2015; York et al., 2016). In infected cells, A3G inhibits viral replication through the specific deamination of dCs in viral minus-strand DNA, thus resulting in massive G-to-A hypermutation of the nascent viral DNA (vDNA) genome during reverse transcription (Lecossier et al., 2003; Zhang et al., 2003; Suspene et al., 2004). The A3G-induced hypermutation is observed as a discrete “all or nothing” phenomenon (Armitage et al., 2012). In addition, A3G directly blocks reverse transcriptase (RT) elongation in a deaminase-independent manner (Guo et al., 2006, 2007; Iwatani et al., 2007; Bishop et al., 2008; Gillick et al., 2013; Chaurasiya et al., 2014) and interferes with the integration of proviral DNA into the host chromosome (Luo et al., 2007; Mbisa et al., 2007, 2010). These cooperative molecular mechanisms are likely to be important in maximizing the anti-HIV-1 activity of A3G. Nevertheless, several studies have shown that the enzymatic activity of A3G is closely correlated with the potential to highly inhibit *vif*-deficient HIV-1 replication (Mangeat et al., 2003; Navarro et al., 2005; Iwatani et al., 2006; Browne et al., 2009). In contrast to understanding of deaminase-independent mechanisms, the details of deaminase-dependent mechanisms, in which A3G inhibits *vif*-deficient HIV-1 replication, are not fully understood.

### Unique Features of A3G-Mediated Deamination

The N-terminal and C-terminal domains (NTD and CTD, respectively) of A3G both contain Zn coordinate motifs ((H/C)xE(x)_23-28_PCxxC; Wedekind et al., 2003; Conticello et al., 2005). The A3G CTD is catalytically active, whereas its NTD has no enzymatic activity but exhibits strong binding to ssDNA and RNA (Hache et al., 2005; Navarro et al., 2005; Iwatani et al., 2006). During the reverse transcription of *vif*-deficient HIV-1, A3G preferentially deaminates the second dC of 5′-CC dinucleotide sites in the newly synthesized viral minus-stranded ssDNA (Harris et al., 2003; Mangeat et al., 2003; Zhang et al., 2003; Yu Q. et al., 2004). This dinucleotide preference is unique among A3 family proteins (Hultquist et al., 2011; Rathore et al., 2013). This deamination occurs more efficiently at the dC close to the 5′-end of ssDNA and less efficiently at the last ∼30 nt of the 3′ ssDNA end, the so-called dead zone (Chelico et al., 2006, 2008). Therefore, it is likely that A3G more efficiently catalyzes the deamination of ssDNA when the A3G CTD is oriented toward the 5′ ssDNA end, and the A3G NTD restricts access of the CTD to the dead zone (Chelico et al., 2010; Shlyakhtenko et al., 2015). Furthermore, the deamination efficacy decreases with decreasing ssDNA length (Chelico et al., 2006), thus probably reflecting the infrequent orientation of the A3G CTD toward the 5′ ssDNA end (Shlyakhtenko et al., 2015).

### Deaminase-Dependent Antiviral Mechanisms

#### Error Catastrophe

**FIGURE 1:**
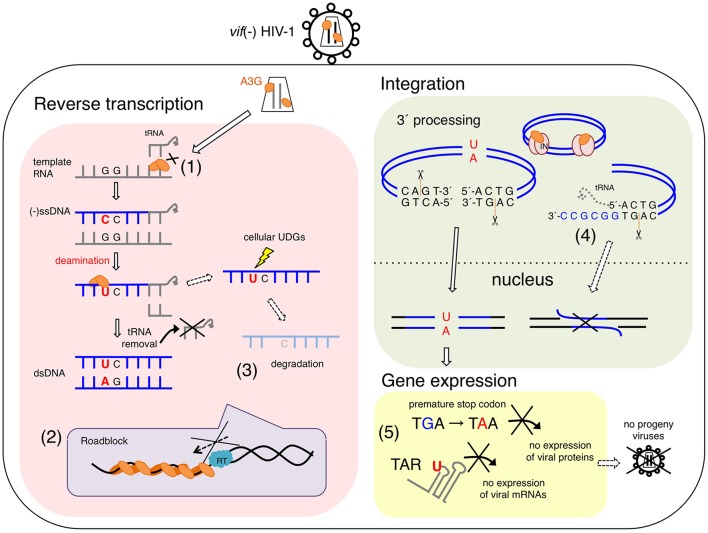
**Proposed antiviral mechanisms of APOBEC3G (A3G) in *vif*-deficient HIV-1-infected cells**. A3G potentially blocks several processes of *vif*-deficient HIV-1 [*vif*(–) HIV-1] replication in a infected cell. (1) A3G interferes with the annealing of the tRNA primer for reverse transcription. (2) A3G physically blocks reverse transcription elongation by binding to RNA and/or ssDNA templates, referred to as a “roadblock” mechanism. A3G oligomerization is closely associated with the efficiency of this action. (3) A3G-induced C-to-U mutations trigger the degradation of reverse transcripts by cellular uracil DNA glycosylases (UNG1, etc.). (3) Direct interaction of A3G with HIV-1 integrase blocks the formation of the replication-competent pre-integration DNA complex. (4) A3G decreases the efficiency and specificity of tRNA primer removal, thereby producing a poor substrate for strand transfer and integration. (5) dC-to-dU conversions in viral (–)ssDNA cause G-to-A hypermutations in progeny viral genomes, thereby leading to viral replication failure, called “error catastrophe.” In part, dC-to-dU mutations in the trans-activation response (TAR) element result in an early block of HIV-1 transcription.

APOBEC3G deaminase activity is crucial for its antiviral activity and restriction of *vif*-deficient HIV-1 replication (Mangeat et al., 2003; Navarro et al., 2005; Iwatani et al., 2006; Browne et al., 2009). An experimental-mathematical study estimated that 99.3% of the antiviral effect of A3G is dependent on its deaminase activity (Kobayashi et al., 2014) (**Figure 1**). Many reports have consistently supported the presumable deaminase-dependent mechanism in which massive A3G-mediated hypermutations in viral reverse transcripts cause lethal mutational loads that terminate progeny virus production and subsequent virus propagation (Harris et al., 2003; Lecossier et al., 2003; Mangeat et al., 2003; Zhang et al., 2003; Suspene et al., 2004; Rawson et al., 2015). This mechanism has previously been described as the error catastrophe mechanism (Crotty et al., 2001; Eigen, 2002; Graci and Cameron, 2002). The mutations introduced in the viral genome, to a certain threshold, lead to sequence diversification, thus enabling adaptation to environmental changes. In contrast, massive amounts of mutations caused by mutagens lead to viral replication failure, called error catastrophe. A3G excessively converts dC to dU in the vDNA of *vif*-deficient HIV-1, thus resulting in G-to-A hypermutations in the viral integrated genomes. These mutations include substitutions of tryptophan codons to in-frame premature stop codons and/or may introduce amino acid changes lethal for viral replication. A3G probably hinders functional viral protein expression and progeny virus production (Pace et al., 2006) (**Figure 1**). A recent study has demonstrated that the introduction of C-to-U mutations in the trans-activation response (TAR) element, a key regulation factor of HIV-1 transcription elongation, results in an early block of viral gene expression (Nowarski et al., 2014) (**Figure 1**).

APOBEC3G-induced G-to-A hypermutations that block viral replication have also been detected in the proviral genomes of infected patients, probably because natural variants of Vif do not completely neutralize A3G and other A3 family proteins (Janini et al., 2001; Kieffer et al., 2005; Simon et al., 2005). Individuals with low to undetectable plasma HIV-1 RNA levels, referred to as “elite controllers,” have frequent G-to-A hypermutations (Gandhi et al., 2008). Therefore, A3G is also presumably involved in the production of defective viruses *in vivo* (Pace et al., 2006; Armitage et al., 2012; Eyzaguirre et al., 2013; Krisko et al., 2013; Sato et al., 2014; Delviks-Frankenberry et al., 2016). However, the incomplete neutralization of A3 family proteins by Vif might result in sequence diversification *in vivo* (Simon et al., 2005; Kim et al., 2010, 2014; Sato et al., 2014; Alteri et al., 2015).

#### Degradation of Uracilated DNA

APOBEC3G, compared with catalytically inactive A3G, decreases the copy number of reverse transcripts in the early phases of infection (Anderson and Hope, 2008; Bishop et al., 2008). In addition, before A3G was identified as a Vif-related cellular factor, von Schwedler et al. (1993) had reported that levels of the reverse transcripts of *vif*-deficient HIV-1 are decreased in newly infected cells when the virus is produced from non-permissive cell lines (currently known as cell lines expressing high amounts of A3G). Thus, it was initially proposed that A3G-induced C-to-U mutations in nascent reverse transcripts might trigger the degradation of reverse transcripts by cellular uracil DNA glycosylases (UDGs), such as nuclear UNG2 and SMUG1 (Harris et al., 2003) (**Figure 1**). The UDG-mediated removal of uracil bases from reverse transcripts might result in the digestion of DNA products at the abasic site by apurinic/apyrimidinic endonuclease. One study further supporting this possibility has shown that the antiviral activity of A3G is partially affected by the UNG2 inhibitor (Ugi) and siRNA specific to UNG2 in virus-producing cells but not in target cells (Yang et al., 2007). However, other studies have shown that UNG2 and SMUG1 are dispensable for the antiviral activity of A3G: A3G-mediated antiviral activity is not changed by Ugi expression (Kaiser and Emerman, 2006; Mbisa et al., 2007; Langlois and Neuberger, 2008), and A3G activity has been observed in Epstein-Barr virus-transformed B-cell lines derived from a *UNG2*^–/–^ patient (Kaiser and Emerman, 2006) and in a SMUG1-deficient avian cell line, with or without exogenous Ugi expression (Langlois and Neuberger, 2008). More recently, two studies have shown involvement of uracilated vDNAs in their chromosomal integration during infection of human cells that contain high levels of dUTP. Yan et al. (2011) have reported that the uracilated vDNA protected it from autointegration, which resulted in facilitating chromosomal integration and viral replication. In contrast, the other study by Hansen et al. (2016) indicated that heavily uracilated vDNAs in monocyte-derived macrophages, not in T-lymphocytes, were not efficiently integrated into chromosomal DNA due to their UNG2-dependent degradation in the nucleus. These data suggest different fate of uracilated vDNA between cytoplasm and nucleus during HIV-1 infection. In addition, because the deaminase-dependent antiviral mechanism has been observed in a variety of cell types, unidentified cellular factors might determine the fate of vDNA containing A3G-induced uracil. Therefore, additional studies are required to determine whether other cellular uracil DNA repair enzymes beyond UNG2 and SMUG1, are involved in the degradation of nascent reverse transcripts.

### Deaminase-Independent Antiviral Mechanisms

Although A3G-mediated deamination was initially proposed to be the sole mechanism of the antiviral activity against *vif*-deficient HIV-1, subsequent studies have demonstrated that other mechanisms are also involved in the inhibition of viral replication. In addition, the enzymatic activity of A3F is not absolutely required for its inhibitory effect on *vif*-deficient HIV-1 replication (Holmes et al., 2007; Luo et al., 2007; Mbisa et al., 2010). Furthermore, a deaminase activity-deficient A3G mutant blocks the replication of HIV-1, mouse mammary tumor virus, and murine leukemia virus, to a certain extent (Okeoma et al., 2007; Belanger et al., 2013), thus suggesting the broad specificity of antiviral activity in terms of the deaminase-independent mechanism.

Initially, Guo et al. (2006, 2007) suggested that A3G might interfere with tRNA^Lys3^ primer placement in viral reverse transcription, in a manner independent of A3G-mediated deamination (**Figure 1**). However, such inhibition of primer annealing has not been observed in other studies (Iwatani et al., 2007; Bishop et al., 2008). Instead, the inhibition of HIV-1 RT elongation has been demonstrated by using *in vitro* and *in vivo* systems (Iwatani et al., 2007; Bishop et al., 2008; Adolph et al., 2013; Belanger et al., 2013) (**Figure 1**). It has been suggested that the inhibitory effect reflects the following unique biochemical characteristics of A3G: (1) A3G protein exhibits high affinity binding specifically to single-stranded polynucleotides, such as ssDNA and RNA (Iwatani et al., 2006; Polevoda et al., 2015); (2) A3G, compared with RT, exhibits significantly higher binding affinity for polynucleotides, although A3G shows similar or slightly less binding affinity for ssDNA than the NC (Iwatani et al., 2006; Darlix et al., 2011); (3) A3G mediates homo-oligomerization in a dose-dependent manner in the presence of ssDNA or RNA, whereas A3G forms monomers, dimers, and tetramers in the absence of these polynucleotides (Wedekind et al., 2006; Salter et al., 2009); and (4) A3G initially binds ssDNA with rapid on-off rates and subsequently converts to a slow dissociation mode after homo-oligomerization (Chaurasiya et al., 2014). Therefore, A3G probably inhibits reverse transcription by tightly binding to the ssDNA or RNA template, thus forming a roadblock that physically obstructs viral DNA synthesis (Iwatani et al., 2007; Adolph et al., 2013; Chaurasiya et al., 2014) (**Figure 1**). This deaminase-independent mechanism might increase the availability of ssDNA for deamination by A3G (Adolph et al., 2013; Chaurasiya et al., 2014), thereby resulting in cooperative effects between deaminase-dependent and deaminase-independent mechanisms.

A3G-mediated inhibition of plus-strand DNA transfer and integration has also been observed (Mbisa et al., 2007, 2010) (**Figure 1**). A3G decreases the efficiency and specificity of tRNA processing and removal during reverse transcription, thereby producing aberrant viral DNA ends defective for efficient plus-strand transfer and integration. Interestingly, it has been reported that A3F exerts an inhibitory effect on viral DNA integration, although its mechanism differs from that of A3G; A3F prevents integration by its binding to the double-stranded DNA of the proviral DNA ends (Mbisa et al., 2010). In contrast to the competition of nucleic acid interactions between A3G and RT/integrase, direct interactions of the A3G protein with HIV-1 RT (Wang et al., 2012) or integrase (Luo et al., 2007) (**Figure 1**) have been reported to be a deaminase-independent mechanism, although the molecular mechanism underlying the specific affinity of A3G for a variety of retroviral RTs and integrases remains unclear. This may be associated with a loss of the reverse transcription complex structure in newly infected cells when A3G coexists with RT (Carr et al., 2006).

### Structural Basis of Antiviral Mechanisms

Recent progress in determining the A3G protein structure has enhanced the current understanding of A3G-mediated antiviral mechanisms, particularly interactions between nucleotides and A3G. First, three-dimensional structures of the A3G CTD were determined by using NMR spectroscopy (Chen et al., 2008; Furukawa et al., 2009; Harjes et al., 2009) and X-ray crystallography (Holden et al., 2008; Shandilya et al., 2010; Li et al., 2012; Lu et al., 2015). Although the structure of the A3G CTD/ssDNA complex has not yet been determined experimentally, four different structural models of the ssDNA-bound A3G CTD have been proposed to explain how A3G recognizes the ssDNA substrate. The first ssDNA-bound model shows the nearly vertical orientation of ssDNA relative to helices α2 and α3 of the A3G CTD along a cleft around the Zn-coordination center (Zn-center pocket; “brim” model) (Chen et al., 2008). The second model suggests that ssDNA binds to the Zn-center pocket with the ssDNA crossed over the cleft seen in the brim model (“kinked” model; Holden et al., 2008). The third model resembles the brim model, although in this model, helices α2 and α3 are involved in ssDNA binding to a greater extent (“straight” model; Furukawa et al., 2009). The recently proposed fourth model based on the crystal structure of the A3G CTD-CTD dimer is a hybrid model of the kinked and brim models (Lu et al., 2015). Nevertheless, it remains inconclusive which ssDNA substrate-binding model is appropriate for deamination catalysis.

**FIGURE 2:**
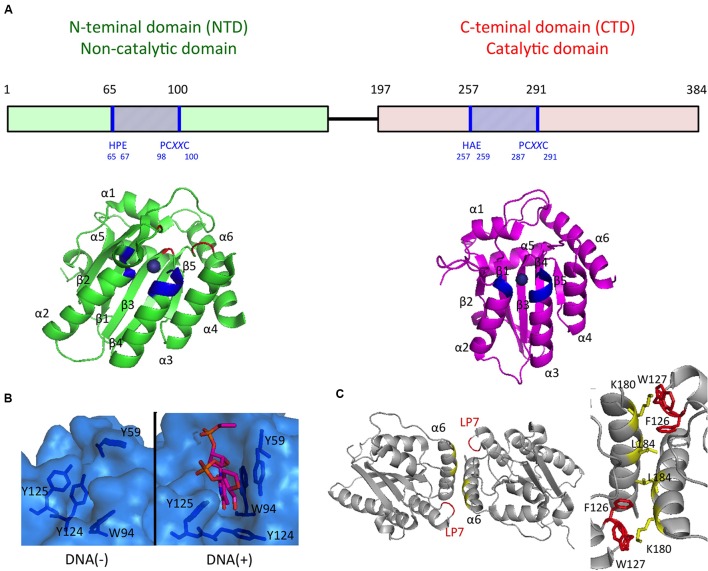
**APOBEC3G domain structures important for the antiviral activities**. **(A)** Schematics of the A3G domain primary sequence and the domain structures are shown in the Upper and Lower, respectively. Zn-coordination motifs and the main DNA/RNA binding sites surrounding Zn are blue and red, respectively. **(B)** The surface representation of the DNA/RNA binding site in the absence (Left) or presence (Right) of a ssDNA substrate. **(C)** Structure of the rhesus A3G-NTD dimer (Left) and key residues involved in dimerization interfaces (Right).

Structures of the highly insoluble A3G NTD protein have recently been determined by using NMR spectroscopy (Kouno et al., 2015) and X-ray crystallography (Xiao et al., 2016). The crystal structure of the A3G NTD, derived from the rhesus macaque (*Macaca mulatta*) protein, reveals a detailed structural mechanism illustrating A3G dimerization and the interaction between the A3G NTD and ssDNA (Xiao et al., 2016). The structural data suggest that ssDNA binding to the A3G NTD changes the conformation of the loops around the Zn-center pocket and Y124 in loop7, thus functioning as a “molecular switch” that regulates the opened/closed status of the Zn-center pocket. The structure also indicates that the dimerization interfaces of the A3G NTD dimer provide a large positively charged surface, including the Zn-center pocket, thereby resulting in the formation of a high affinity surface toward the ssDNA or RNA (**Figure 2**). These structural features are consistent with results of previous biochemical studies suggesting that the NTD-NTD interaction is crucial for A3G oligomerization, nucleic acid binding, and the antiviral activity of A3G (Bennett et al., 2008; Huthoff et al., 2009; Chelico et al., 2010; Shandilya et al., 2010; Belanger et al., 2013; Chaurasiya et al., 2014).

## Conclusion

Recent evidence suggests that A3G executes potent antiviral activity through cooperative deaminase-dependent and deaminase-independent mechanisms. Undoubtedly, the enzymatic activity of A3G is closely correlated with the potential to inhibit *vif*-deficient HIV-1 replication. However, it remains unclear how the A3G-mediated deamination event is linked to the A3G-mediated lethal inhibition of viral replication. Further studies of the molecular mechanisms of A3G antiviral activity, particularly for the deaminase-dependent mechanisms, are required, including the careful determination of the fate of uracil-containing viral DNA in newly HIV-1-infected cells.

## Author Contributions

AO and YI analyzed the data and wrote the paper.

## Conflict of Interest Statement

The authors declare that the research was conducted in the absence of any commercial or financial relationships that could be construed as a potential conflict of interest.
